# Public health round-up

**DOI:** 10.2471/BLT.20.011220

**Published:** 2020-12-01

**Measles cases rise**

A baby is immunized at a community health centre in Uganda. According to a new report, measles cases have surged worldwide, with 869 770 cases reported in 2019, the highest reported level in 23 years. The authors of the report cite a failure to vaccinate children on time as the main driver of these trends.

**Figure:**
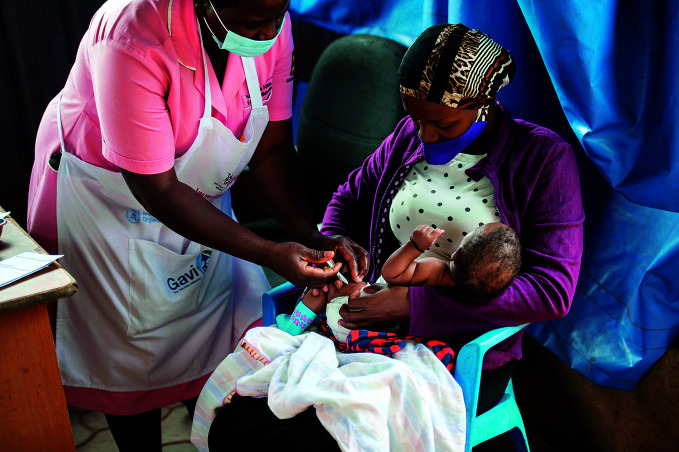
UNICEF / Kalungi Kabuye

## Measles surges

Measles cases have surged worldwide, with 869 770 cases reported in 2019, the highest reported number since 1996.

According to a report published on 12 November by the World Health Organization (WHO) and the United States Centers for Disease Control and Prevention, global measles deaths have also increased, rising to 207 500 deaths in 2019, almost 50% higher than reported deaths in 2016.

After steady progress between 2010 and 2016, the number of reported measles cases steadily increased in 2019. The report’s authors cite a failure to vaccinate children on time with two doses of measles-containing vaccines (MCV1 and MCV2) as the main driver of the increasing trends in cases and deaths.

To control measles and prevent deaths, vaccination coverage rates with MCV1 and MCV2 must reach 95% and be maintained at national and subnational levels. MCV1 coverage has been stagnant globally for more than a decade at around 85%. MCV2 coverage has been steadily increasing but is still only at 71%.

Although reported cases of measles are lower in 2020, efforts to control COVID-19 have resulted in disruptions in vaccination and hampered efforts to minimize measles outbreaks.

“These data send a clear message that we are failing to protect children from measles in every region of the world,” said WHO Director-General Tedros Adhanom Ghebreyesus. “We must collectively work to support countries and engage communities to reach everyone, everywhere with measles vaccine and stop this deadly virus.”

https://bit.ly/2IuyJ9h

## Polio PHEIC

The risk of international spread of wild poliovirus may be at the highest level since 2014 and the COVID-19 pandemic is seriously hampering polio eradication programme and outbreak control efforts.

These were among the conclusions of the Emergency Committee under the International Health Regulations (2005) on the international spread of poliovirus which was convened on 14 October and reported its proceedings on 22 October.

While no wild poliovirus cases have been detected outside of Afghanistan and Pakistan since 2016, in the view of the committee, the spread of wild poliovirus between the two countries and the increased vulnerability of countries where routine immunization and polio prevention activities are being adversely affected by the COVID-19 pandemic represent significant risks.

The committee unanimously agreed that the risk of international spread of poliovirus continues to represent a Public Health Emergency of International Concern (PHEIC) and recommended the extension of the PHEIC for a further three months.

The committee also reviewed the data on circulating vaccine-derived polioviruses and expressed concern about continued international spread, noting new outbreaks in Guinea, South Sudan and Sudan.

The committee urged countries and donors to maintain funding of polio eradication activities, judging the potential for reversal of progress to be high.

https://bit.ly/3kkz1fN

## New SARS-CoV-2 strain

SARS-CoV-2 infections associated with farmed minks have been reported in Denmark. As of 5 November, 214 human cases had been reported, including 12 infections with a unique variant of the pathogen.

All 12 cases were identified in September 2020 in North Jutland, Denmark. The people infected ranged from 7 to 79 years of age, eight had a link to the mink farming industry and four were from the local community.

Initial observations suggest that the clinical presentation, severity and transmission among those infected are similar to those caused by other circulating SARS-CoV-2 viruses.

However, the new strain, referred to as the "cluster 5" variant, has a combination of mutations not previously observed. The implications of the mutations are not yet well understood and will require further study.

https://bit.ly/3nbD2oC

## Regulating COVID-19 products

The International Coalition of Medicines Regulatory Authorities (ICMRA) and WHO committed to working together to expedite equitable access to safe and effective COVID-19-related health products.

Anticipating the potentially imminent roll-out of a large volume of new COVID-19 vaccines and treatments, the institutions have joined forces to uphold and promote rigorous, evidence-based regulation and regulatory alignment across countries.

In a joint statement issued on 6 November, the ICMRA and WHO stressed the need to ensure that existing evidence-based standards of review and oversight are maintained while facilitating expedited patient access to safe and effective medical products.

To achieve these aims, the institutions committed to promoting and supporting prioritization of well-designed, transparent clinical trials that provide robust results, data sharing between regulators in real time to facilitate multi-country approvals, and regulatory agility to expedite regulatory processes.

https://bit.ly/3kk98ge

## HIV prevention progress

A long-acting, injectable antiretroviral drug has been shown to be highly effective in preventing human immunodeficiency virus (HIV) acquisition in women. According to a statement issued by WHO on 9 November, a clinical trial designed to test the safety and efficacy for pre-exposure prophylaxis (PrEP) of cabotegravir (CAB LA) was halted early by the trial Data and Safety Monitoring Board, having reached a positive conclusion.

The outcome of the trial has no bearing on evidence showing that consistently using oral PrEP is highly effective, as has been demonstrated in several trials. Oral daily PrEP remains an effective prevention option for anyone at substantial HIV risk and has been recommended by WHO since 2015. However, even short lapses in taking oral PrEP can reduce protection. CAB LA may be an alternative option for women.

https://bit.ly/38yvWpZ

## Antenatal steroid efficacy

Dexamethasone has been shown to boost the survival of premature babies when given to pregnant women at risk of preterm birth in resource-poor settings.

This is the key finding of WHO ACTION-I, a randomized clinical trial, the results of which were published in the *New England Journal of Medicine* on 23 October. The trial resolves an ongoing controversy about the efficacy of antenatal steroids for improving preterm newborn survival in low-income countries.

The trial was conducted from December 2017–November 2019 and recruited 2852 women and their 3070 babies from 29 secondary and tertiary level hospitals in Bangladesh, India, Kenya, Nigeria and Pakistan.

https://bit.ly/2IoGrRS

**Cover photo**

Midwife Heather Heinrichs examines a baby in the subarctic town of Hay River in Canada’s Northwest Territories.

**Figure:**
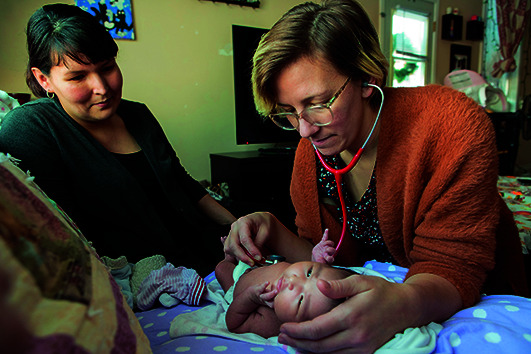
WHO / Christine McNab

## Wikimedia

WHO and the Wikimedia Foundation, the not-for-profit organization that operates Wikipedia and other Wikimedia free knowledge projects, are collaborating to expand public access to timely, reliable information about COVID-19.

Announced on 22 October, the collaboration will make trusted, public health information freely available under the Creative Commons Attribution-ShareAlike licence. People with internet access will be able to access and share WHO infographics, images, videos and other public health assets on Wikimedia Commons, a free digital library.

By making verified information about the pandemic available to more people on one of the world’s most-visited knowledge platforms, the organizations aim to help counter the “infodemic” of COVID-19 misinformation and misrepresentation.

“Equitable access to trusted health information is critical to keeping people safe and informed during the COVID-19 pandemic,” said WHO Director-General Tedros Adhanom Ghebreyesus. "Our new collaboration with the Wikimedia Foundation will increase access to reliable health information from WHO across multiple countries, languages and devices."

https://bit.ly/36nGq93

## Health emergency resolution

At the time of going to press, WHO’s 194 Member States were expected to adopt a resolution to strengthen preparedness for health emergencies at the resumed Seventy-third World Health Assembly.

The draft resolution renews the commitment to better prepare for health emergencies such as COVID-19 through full compliance with the International Health Regulations and urges Member States to invest in health emergency preparedness, improve government and decision-making processes, and enhance institutional capacity and infrastructure for public health.

The World Health Assembly usually takes place in May. Because of the COVID-19 pandemic, this year a reduced World Health Assembly took place on 18–19 May. The resumed Seventy-third World Health Assembly took place virtually from 9–14 November.

https://bit.ly/3nciQ5T

**Looking ahead**

December 3 – International Day of Persons with Disabilities. https://bit.ly/36oZgNh

December 3–4 – General Assembly of the United Nations special session on COVID-19. https://bit.ly/32xpmMX

December 12 – International Universal Health Coverage Day. https://bit.ly/2UitwUq

